# Case Report: Genomic and clinical insights into MYBPC3-related hypertrophic cardiomyopathy in Ecuadorian patients: implications for sudden cardiac death risk

**DOI:** 10.3389/fcvm.2025.1693244

**Published:** 2026-01-21

**Authors:** Elius Paz-Cruz, Patricia Guevara-Ramírez, Rafael Tamayo-Trujillo, Viviana A. Ruiz-Pozo, Santiago Cadena-Ullauri, Rita Ibarra-Castillo, José Luis Laso-Bayas, Leonel Meza-Chico, Alejandro Cabrera-Andrade, Ana Karina Zambrano

**Affiliations:** 1 Universidad UTE, Centro de Investigación Genética y Genómica, Facultad de Ciencias de la Salud Eugenio Espejo, Quito, Ecuador; 2 Clinical Cardiac Electrophysiologist, Quito, Ecuador; 3 Grupo de Bio-Quimioinformática, Universidad de Las Américas, Quito, Ecuador; 4 Carrera de Enfermería, Facultad de Ciencias de la Salud, Universidad de Las Américas, Quito, Ecuador

**Keywords:** cardiovascular disease, Ecuadorian, genetics, genomics, healthcare, hypertrophic cardiomyopathy

## Abstract

Hypertrophic cardiomyopathy (HCM) is the most common inherited cardiac disease and a leading cause of sudden cardiac death (SCD) in young adults and athletes. It exhibits marked clinical variability, which may be influenced by genetic background and environmental factors. Although *MYBPC3* is the most frequently implicated gene, data from Latin American and admixed populations remain scarce. In this study, we describe three unrelated Ecuadorian patients with clinically diagnosed HCM who harbored *MYBPC3* variants. Two patients carried likely pathogenic mutations (p.Glu258Lys and p.His875Profs*8), while novel missense variants (p.Ala536Pro and p.Thr274Met) were identified as variants of uncertain significance (VUS). Additional variants were detected in *TTN*, *MYLK2*, *RYR1*, *SDHA*, *APOB*, and *JPH2*, but given their classification as VUS or a lack of association with HCM, they are described only as incidental findings. An ancestry analysis revealed heterogeneous contributions of Native American, European, and African backgrounds, reflecting the admixed composition of the Ecuadorian population. This case series underscores the phenotypic heterogeneity of HCM, even among patients with *MYBPC3* variants, and highlights the importance of genomic testing in underrepresented populations to improve diagnosis, family screening, and SCD risk stratification.

## Introduction

Hypertrophic cardiomyopathy (HCM) is one of the most common autosomal dominant inherited cardiac diseases, with an estimated prevalence of 1 in 200–500 individuals in the general population (1). Recent estimates suggest that approximately 20 million people are affected worldwide, although only approximately 10% of cases are clinically recognized (2). The diagnosis of HCM is based on the presence of unexplained left ventricular hypertrophy in a non-dilated ventricle, typically identified by echocardiography or cardiac magnetic resonance imaging (3). HCM represents one of the leading causes of sudden cardiac death (SCD), particularly in young adults and athletes (4). Despite advances in imaging and risk stratification, the clinical course of HCM remains highly variable, ranging from asymptomatic individuals with near-normal life expectancy to patients who develop severe heart failure, arrhythmias, or SCD (3).

Molecular genetics has transformed the understanding of HCM. Pathogenic variants in genes encoding sarcomeric proteins account for up to 60% of cases, with *MYBPC3* and *MYH7* being the most frequently implicated (5). Variants in *MYBPC3* are especially common and include both truncating and missense changes. Truncating variants are typically associated with haploinsufficiency through nonsense-mediated RNA decay (NMD) and ubiquitin–proteasome system (UPS) degradation, while the clinical impact of missense variants is more heterogeneous (5–7). The wide phenotypic spectrum of HCM likely reflects the combined effects of primary sarcomeric variants, variable penetrance, and non-genetic influences (8).

Although most genetic studies have been conducted in North American and European cohorts, Latin American and admixed populations remain underrepresented in the literature (9). The genetic diversity of these populations, shaped by varying proportions of Native American, European, and African ancestry, provides a unique context to explore the molecular and phenotypic variability of HCM. Characterizing these cohorts is crucial to improve risk stratification, expand the catalog of variants with clinical relevance, and enhance the understanding of ancestry-related contributions to disease expression (9, 10).

In this study, we describe the clinical and genomic evaluation of three unrelated Ecuadorian patients with HCM. This case series aims to explore genotype–phenotype correlations, assess the potential contribution of additional cardiomyopathy-associated variants, and consider the role of genetic ancestry in an admixed population. By doing so, we seek to expand the understanding of HCM in underrepresented populations and highlight the value of genomic testing for diagnosis, family screening, and SCD risk stratification.

## Case presentation

Three unrelated Ecuadorian patients with HCM and family histories suggestive of hereditary cardiac disease were included in this study. The diagnosis of HCM was established clinically, without initial genetic confirmation. Clinical diagnosis was based on symptoms, family history, and echocardiographic findings following established adult and pediatric criteria. The clinical diagnosis of HCM prompted genomic testing and ancestry analysis. To carry out these analyses, peripheral blood samples were collected, and genomic DNA was extracted and quantified following standard protocols. DNA quality and concentration were verified fluorometrically. Next-generation sequencing (NGS) was performed at the Centro de Investigación Genética y Genómica (CIGG), UTE University, using the TruSight Cardio panel (Illumina), which analyzes 174 genes associated with inherited cardiac disorders. Sequencing was conducted on a MiSeq platform, and variant interpretation followed ACMG guidelines, integrating population frequency data, in silico predictions, and genotype–phenotype correlation. The interpretation of the genomic findings was performed jointly by cardiologists and geneticists to ensure accurate clinical–genetic correlation. In addition, genetic ancestry was evaluated using a validated panel of 46 ancestry-informative insertion/deletion markers (AIMs-InDels). Genotyping was performed by using PCR and capillary electrophoresis, and ancestry inference was carried out with STRUCTURE software (v2.3.4), yielding proportional estimates of Native American, European, and African ancestry.

## Subject 1

**Figure 2:**
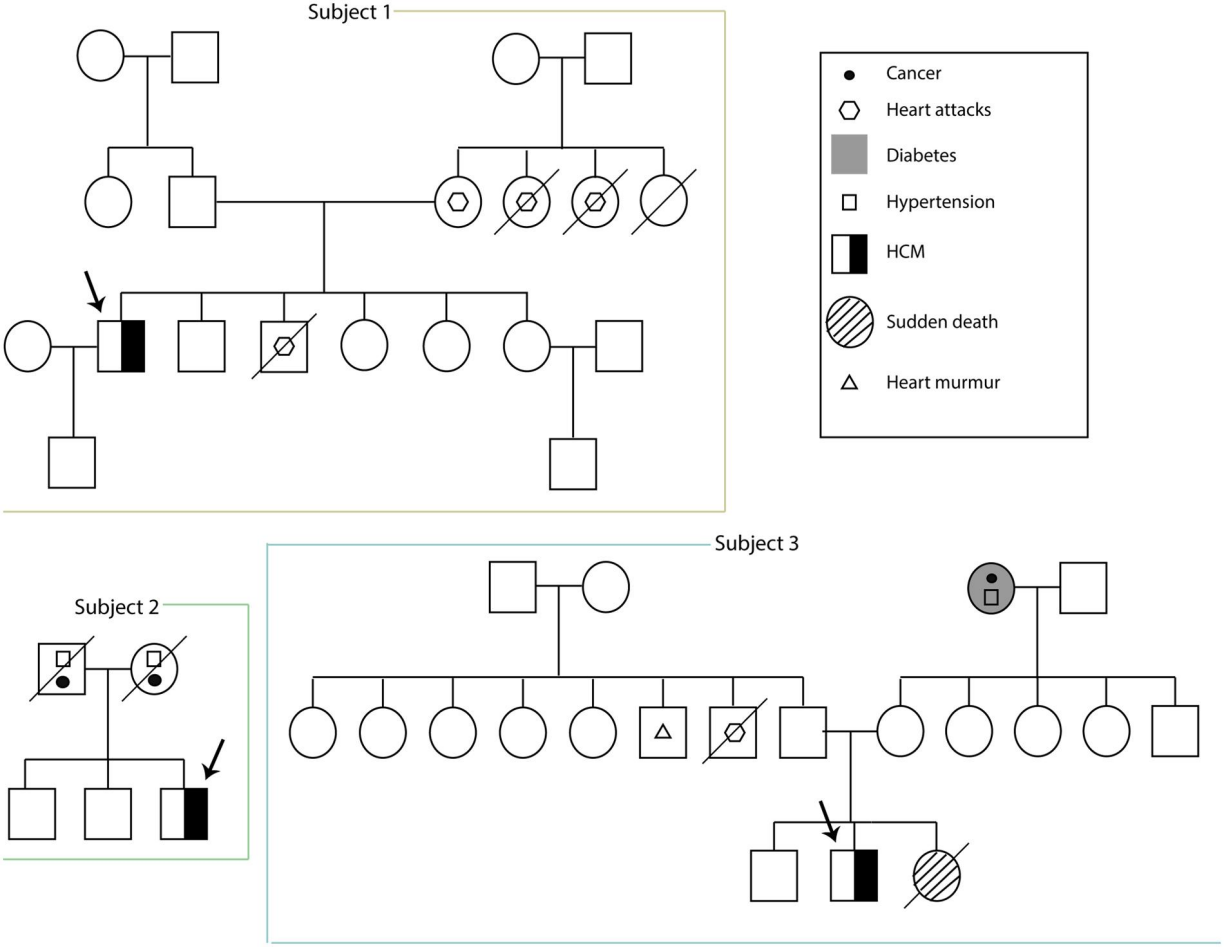
Familial pedigrees of three unrelated male patients (subjects 1–3) with HCM. The proband in each family is indicated by an arrow.

A 44-year-old male from Guaranda was diagnosed with HCM in 2014. His medical history included long-term atenolol therapy and implantation of an implantable cardioverter-defibrillator (ICD) in 2019 for primary prevention, based on an estimated 5-year SCD risk of 3.9% according to the European Society of Cardiology (ESC) risk model. Transthoracic echocardiography demonstrated asymmetric septal hypertrophy, with a maximal interventricular septal thickness of 24 mm, dynamic left ventricular outflow tract obstruction (maximum gradient 40 mmHg), and systolic anterior motion of the mitral valve. Left ventricular systolic function was preserved (ejection fraction 70%), with evidence of grade II diastolic dysfunction. Family history was notable for first-degree relatives with myocardial infarction (Figure 2).

Genetic testing revealed a likely pathogenic variant in *MYBPC3* (p.Glu258Lys). Additional findings included two variants of uncertain significance (VUS) in *TTN* (p.Arg3224Gly) and *MYLK2* (p.Gln525Arg) (Table 1). The ancestry profile demonstrated a predominant Native American component (46.7%), with European (38.5%) and African (14.8%) contributions.

**Table 1:** Description of the genetic variants identified in patients diagnosed with hypertrophic cardiomyopathy (HCM). Variants were classified according to ACMG-AMP guidelines.

Subject	Age (years)	Gender	Gene	Genetic variant c.notation/p.notation	Consequence^a^	Classification reference SNP	ACMG guidelines (1, 11)
1	44	Male	*MYBPC3*	NM_000256.3	Missense	Likely pathogenic	PS4, PP1, PS3, PM2, PP3, PP5
				c.772G>A			
				p.(Glu258Lys)		rs397516074	
			*TTN*	NM_001267550.2	Missense	VUS	PM2
				c.9670A>G			
				p.(Arg3224Gly)			
			*MYLK2*	NM_033118.3	Missense	VUS	PM2, BP4
				c. 1574A>G			
				p.(Gln525Arg)			
2	57	Male	*MYBPC3*	NM_000256.3	Missense	VUS	PM2, PP3
				c.1606G>C			
				p.(Ala536Pro)			
			*TTN*	NM_001267550.2	Missense	VUS	PM2
				c.22148A>G			
				p.(Asn7383Ser)			
			*RYR1*	NM_000540.2	Missense	VUS	PM2, PP2
				c.9494G>A			
				p.(Cys3165 Tyr)			
3	17	Male	*MYBPC3*	NM_000256.3	Frameshift Indels	Likely pathogenic	PVS1, PM2
				c.2624_2625del			
				p.(His875Profs*8)			
			*MYBPC3*	NM_000256.3	Missense	VUS	PM2,PP3
				c.821C>T			
				p.(Thr274Met)			
			*SDHA*	NM_004168.3	Frameshift Indels	VUS	PVS1
				c.1945_1946del			
				p.(Leu649Glufs*4)			
			*APOB*	NM_000384.2	Missense	VUS	PM2, BP6
				c.3443T>A			
				p.(Leul148His)			
			*JPH2*	NM_020433.4	Missense	VUS	PM2, BP4
				c. 1615G>A			
				p. (Ala539Thr)			

a All variants are heterozygous; ^1^*PVS1*: Predicted null variant (e.g., nonsense and frameshift) in a gene where loss of function is a known mechanism of disease; *PS4*: Prevalence of the variant in affected individuals significantly increased compared with controls; *PP1*: Cosegregation with disease in multiple affected family members; *PS3*: Functional studies supportive of a damaging effect on the gene or gene product; *PM2*: Absent or rare in population databases; *PP3*: Multiple computational lines of evidence support a deleterious effect; *PP5*: Reputable source reports the variant as pathogenic; *BP4*: Computational evidence supports a benign effect; *BP6*: Reputable source reports the variant as benign; *PP2*: Missense variant in a gene with a low rate of benign missense variation and where missense variants are a common mechanism of disease.

## Subject 2

**Figure 1:**
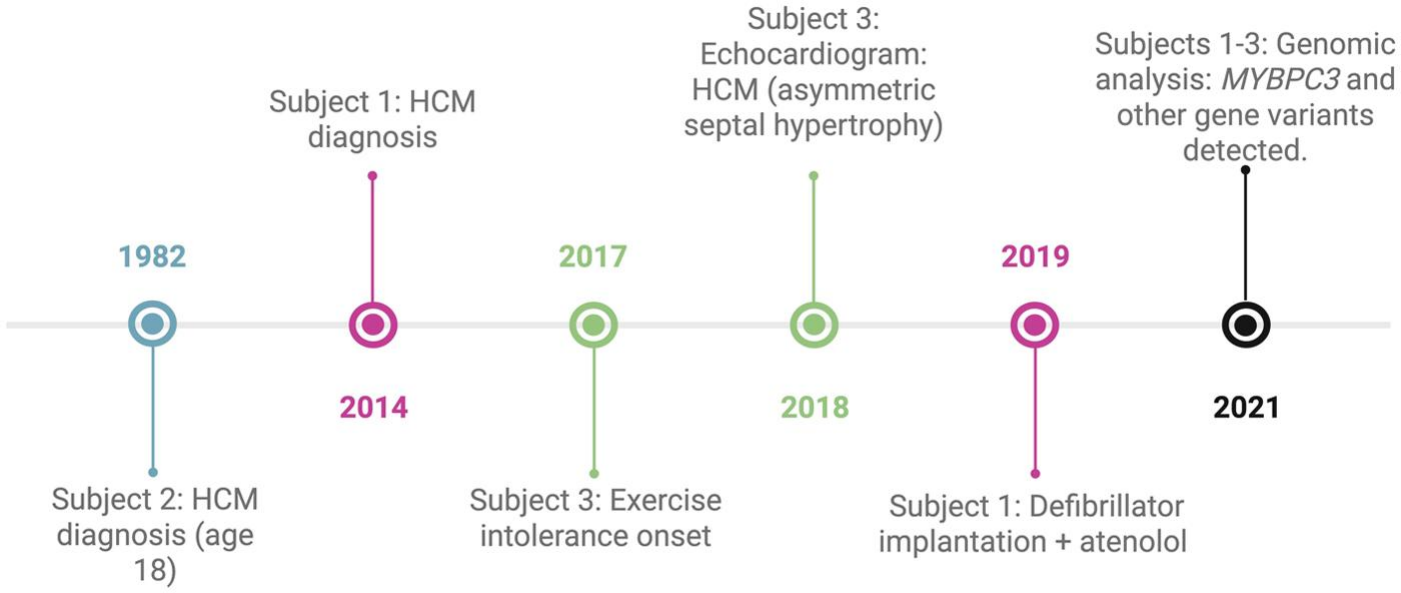
Clinical and genomic timelines of three Ecuadorian patients with HCM. Each case is represented by a distinct color: pink for subject 1, blue for subject 2, green for subject 3, and black for events shared across all three cases.

A 57-year-old male from Zaruma was diagnosed with HCM at age 18 (Figure 1). He reported no regular pharmacological treatment. His family history included parental hypertension and cancer (Figure 2).

Genetic testing identified only variants of uncertain significance: *MYBPC3* (p.Ala536Pro), *TTN* (p.Asn7383Ser), and *RYR1* (p.Cys3165Tyr) (Table 1). Furthermore, an ancestry analysis showed a predominance of European ancestry (56.2%), with Native American (24.5%) and African (19.3%) components.

## Subject 3

A 19-year-old male from Ibarra presented with a history of exertional intolerance since childhood, characterized by fatigue and presyncope first documented in 2017 (Figure 1). Perinatal history included late preterm birth and neonatal jaundice secondary to maternal preeclampsia. Family history was notable for a sister who died at three months of age from presumed sudden infant death syndrome (SIDS), although this cannot be classified as confirmed sudden cardiac death because of the lack of autopsy data. Additional relatives had cardiovascular conditions, including a paternal uncle with myocardial infarction at 58, another with a heart murmur, and a grandmother with hypertension (Figure 2).

A transthoracic echocardiogram performed in 2018 showed asymmetric interventricular septal hypertrophy, with septal thickness evaluated according to pediatric age- and body-size–adjusted reference standards, which at the time of the study were interpreted by the pediatric cardiologist as consistent with HCM. Left ventricular systolic function was preserved. The study also identified a bicuspid aortic valve with preserved opening and mild functional impairment. No additional structural abnormalities were observed. These findings supported the diagnosis of non-obstructive HCM with asymmetric septal hypertrophy and an associated bicuspid aortic valve.

A genomic analysis identified a likely pathogenic frameshift variant in *MYBPC3* (p.His875Profs*8), together with a VUS in the same gene (p.Thr274Met). Additional VUS were detected in *SDHA* (p.Leu649GlufsTer4), *APOB* (p.Leu1148His), and *JPH2* (p.Ala539Thr) (Table 1). An ancestry analysis revealed a predominant Native American component (69.6%), with European (22.4%) and African (8.0%) contributions.

## Discussion

This report describes the clinical and genomic features of three unrelated Ecuadorian patients with HCM carrying variants in *MYBPC3*, the gene most frequently implicated in this disorder. Two variants, p.Glu258Lys and p.His875Profs*8, were classified as likely pathogenic, whereas p.Ala536Pro and p.Thr274Met were classified as VUS. Together, these findings expand the molecular spectrum of *MYBPC3*-related HCM and represent the first characterization of such cases in Ecuador, underscoring the importance of genomic studies in underrepresented admixed populations.

Consistent with previous reports, *MYBPC3* variants in this series were associated with marked phenotypic variability (12–14). Such heterogeneity was observed even in monozygotic twins carrying the same pathogenic *MYBPC3* variant (p.G263Ter), who exhibited distinct degrees of penetrance and clinical severity (15). All three probands in our cohort were male and developed HCM during adolescence or early adulthood. Two displayed high-risk clinical features, ICD implantation in Subject 1 and a family history of SCD in Subject 3, highlighting the potential for malignant arrhythmias even within a small cohort. This variability among carriers of pathogenic *MYBPC3* variants likely reflects the combined influence of genetic, environmental, and modifier factors on disease expression and progression (12–14).

The *MYBPC3* gene encodes cardiac myosin-binding protein C (cMyBP-C), a sarcomeric protein that interacts with actin, myosin, and titin and plays a critical role in regulating myocardial contractility. Truncating *MYBPC3* variants typically introduce premature stop codons, leading to reduced protein levels through nonsense-mediated RNA decay (NMD) and degradation by the ubiquitin–proteasome system (UPS), resulting in haploinsufficiency (16). In Subject 3, we identified a novel truncating variant, p.His875Profs*8, classified as likely pathogenic and consistent with the proband's phenotype of non-obstructive HCM with asymmetric septal hypertrophy. Similar truncating *MYBPC3* variants have been associated with left ventricular hypertrophy and ventricular tachycardia, supporting haploinsufficiency as the main disease mechanism (16).

In addition to truncating variants, three MYBPC3 missense variants were detected: p.Glu258Lys in Subject 1, p.Ala536Pro in Subject 2, and p.Thr274Met in Subject 3. The p.Glu258Lys variant has been reported in several individuals with HCM and has been classified as pathogenic in multiple studies (17–20), supporting its role in the severe phenotype observed in Subject 1. In contrast, p.Ala536Pro and p.Thr274Met have not been previously described in HCM patients and are currently classified as VUS (21, 22).

With respect to p.Ala536Pro, a substitution at the same residue (p.Ala536Gly) was described in a 13-year-old proband with HCM (23), suggesting functional relevance of this region of *MYBPC3*. Although alanine and proline share similar biochemical properties, substitutions at this residue may still influence protein stability, alter sarcomeric interactions, or affect protein turnover through NMD/UPS pathways. Nevertheless, available evidence is insufficient to establish pathogenicity, and the contribution of p.Ala536Pro to the phenotype of Subject 2 remains uncertain. Additional segregation and functional studies are required to better define its clinical relevance (12).

A similar level of caution applies to p.Thr274Met, detected in Subject 3. Although methionine has biochemical properties that are different from those of threonine, computational models have predicted no major structural or functional impact for this substitution (22). The coexistence of a missense variant with a truncating MYBPC3 mutation raises the possibility of cumulative effects on protein dosage; however, this remains uncertain and cannot be inferred without segregation or functional data.

Variants in additional genes were also identified. Subject 1 harbored *MYLK2* p.Gln525Arg. *MYLK2* encodes myosin light chain kinase 2, a calcium/calmodulin-dependent enzyme that is predominantly expressed in the skeletal muscle and phosphorylates the regulatory light chain of myosin (24). Pathogenic *MYLK2* variants have been reported in association with midventricular hypertrophic cardiomyopathy and cardiac repolarization abnormalities, including inverted T waves on electrocardiogram, as described in carriers of the p.Lys324Glu mutation (25, 26). Although the p.Gln525Arg variant has not been previously described in patients with HCM, its location within a conserved kinase domain raises the possibility of functional relevance (25).

In addition, *TTN* variants were identified in both Subject 1 (p.Arg3224Gly) and Subject 2 (p.Asn7383Ser). The p.Arg3224Gly variant has been previously reported in an individual with ventricular fibrillation (27), while the p.Asn7383Ser variant has not been previously described. *TTN* is essential for sarcomeric structure, providing mechanical stability and acting as a molecular scaffold that coordinates interactions with multiple contractile and signaling proteins (16). Although *TTN* missense variants are common in the general population and most lack diagnostic value in HCM (28), emerging evidence indicates that a small subset of *TTN* missense variants can contribute to disease when supported by segregation and functional data. For example, a recent study in a Chinese HCM pedigree identified a *TTN* missense variant p.Arg6745Cys that segregated with affected family members and was predicted to impair protein function, supporting its pathogenic role (29). These findings suggest that *TTN* missense variants cannot be categorically dismissed (28); however, the variants identified in our patients do not currently meet criteria for pathogenicity or modifier status, and their clinical significance therefore remains uncertain.

In addition, Subject 2 harbored the *RYR1* p.Cys3165Tyr variant, which has not been previously associated with HCM. Pathogenic variants in *RYR1* are classically linked to early-onset myopathies (30). *RYR1* encodes ryanodine receptor 1, a sarcoplasmic reticulum Ca^2+^ release channel that plays a central role in excitation–contraction coupling (31). While its expression is predominant in the skeletal muscle, *RYR1* is also detected in cardiac and vascular tissues, where it contributes to intracellular Ca^2+^ homeostasis (32). Experimental studies have suggested that alterations in *RYR1* expression or function may promote left ventricular hypertrophy by modulating calcium flux and myocardial remodeling (33). The p.Cys3165Tyr variant has not been reported in patients with HCM; however, its localization within a regulatory domain indicates that further investigation would be required to determine whether it has any physiological relevance in the context of this case.

Subject 3 carried the *SDHA* p.Leu649Glufs*4 and APOB p.Leu1148His variants. *SDHA* encodes a subunit of mitochondrial complex II (succinate dehydrogenase), a critical component of oxidative phosphorylation (34). Although *SDHA* is not an established gene for primary sarcomeric HCM, pathogenic *SDHA* variants have been reported in association with cardiomyopathy exhibiting hypertrophic features, most often as part of multisystem mitochondrial disease (34, 35). Notably, the same *SDHA* p.Leu649Glufs*4 variant has been described in a young woman with mitochondrial disease presenting with left ventricular hypertrophy and cardiomyopathy in the setting of compound heterozygous *SDHA* variants (36). These observations support cardiac involvement as a component of SDHA-related disease; however, they do not establish *SDHA* as a cause of isolated HCM. In the present case, the absence of additional clinical or biochemical features suggestive of mitochondrial dysfunction precludes attribution of the HCM phenotype to the *SDHA* variant, and its contribution therefore remains uncertain.

In addition to the *SDHA* variant, Subject 3 also carried *APOB* p.Leu1148His. *APOB* encodes apolipoprotein B, the principal structural component of low-density lipoprotein (37), and pathogenic variants in this gene are primarily associated with familial hypercholesterolemia, leading to elevated LDL cholesterol levels and an increased risk of premature atherosclerotic cardiovascular disease (38). Although *APOB* is not related to sarcomeric HCM and does not contribute to the development of myocardial hypertrophy, *APOB* variants may be clinically relevant by increasing overall cardiovascular risk (39) through dyslipidemia and ischemic mechanisms, which can predispose to adverse cardiac events, including sudden cardiac death, particularly in the presence of underlying structural heart disease (40).

Subject 3 also carried a *JPH2* missense variant (p.Ala539Thr). *JPH2* encodes junctophilin-2, a structural protein essential for the formation of junctional membrane complexes that facilitate excitation–contraction coupling in cardiomyocytes. While *JPH2* is not a canonical sarcomeric gene, a pathogenic JPH2 mutation (p.Thr161Lys) was implicated as a non-sarcomeric candidate gene for HCM in Finnish families, where it was associated with ventricular hypertrophy, arrhythmia, and conduction abnormalities (41).

In this context, Subject 3 carried a complex genetic profile, including a truncating *MYBPC3* variant (p.His875Profs*8), an additional *MYBPC3* missense variant (p.Thr274Met), a truncating *SDHA* variant (p.Leu649Glufs*4), the *APOB* p.Leu1148His variant, and the JPH2 p.Ala539Thr variant. The *MYBPC3* truncating variant represents the most compelling genetic explanation for the HCM phenotype. In contrast, the *SDHA* and *JPH2* variants cannot be assigned a causal or modifying role with the available data and are best interpreted as secondary or incidental findings. The *APOB* p.Leu1148His variant likewise does not support a causal or modifying role in HCM; however, it may represent a relevant comorbidity that could contribute to overall cardiovascular risk in the presence of a confirmed sarcomeric disease substrate. Clarification of the individual or combined relevance of these secondary variants will require segregation analysis and functional studies.

Altogether, our findings illustrate how *MYBPC3* variants, particularly truncating mutations such as p.His875Profs*8, contribute to phenotypic severity and may increase susceptibility to malignant arrhythmias and sudden cardiac death. Additional variants detected in non-sarcomeric or non-HCM genes are reported as part of the broader genetic profile, although their clinical relevance in HCM remains uncertain. Subject 1, who required implantable cardioverter-defibrillator implantation, and Subject 3, with a strong family history of sudden cardiac death, exemplify the critical need for careful surveillance and timely clinical intervention. Collectively, these cases underscore the importance of integrating genomic findings with conventional risk stratification tools to optimize preventive strategies against SCD.

As for ancestry, Subjects 1 and 3 showed a higher proportion of Native American background. HCM has not been shown to have a higher prevalence in any specific ethnic group (16). However, studies have reported that Black patients are diagnosed at a younger age than white patients, despite exhibiting a lower frequency of sarcomeric mutations (42). In our cohort, the admixed ancestry background did not allow establishing a clear association between ethnicity and HCM susceptibility, particularly given the high proportion of European ancestry in Subject 2. Therefore, a more comprehensive characterization of HCM in admixed populations is needed to determine whether ancestry influences disease pathogenesis, severity, or prognosis.

Several limitations in this study should be acknowledged. Further exploration of the family pedigrees was limited by the unavailability or lack of consent from additional relatives. Moreover, access to the complete medical records of the patients was restricted, which prevented a more detailed analysis of clinical evolution, treatment response, and follow-up. Although familial HCM was suspected in all three cases, the absence of confirmed diagnoses in previous generations raises the possibility that some *MYBPC3* variants could represent *de novo* events or show incomplete penetrance. Future cascade genetic testing in extended family members will be essential to clarify segregation and inheritance patterns and refine genetic risk assessment.

## Conclusion

This study provides a clinical and genomic characterization of three unrelated Ecuadorian individuals with hypertrophic cardiomyopathy harboring variants in MYBPC3, as well as additional variants in TTN, MYLK2, RYR1, SDHA, APOB, and JPH2, reinforcing the marked phenotypic heterogeneity of HCM even among patients with variants in the same primary disease gene. The identification of likely pathogenic truncating MYBPC3 variants supports haploinsufficiency, mediated by nonsense-mediated RNA decay and proteasomal degradation, as a central mechanism of disease pathogenesis. In contrast, missense variants and variants detected in non-sarcomeric or non-HCM genes were classified as variants of uncertain significance, underscoring the challenges of variant interpretation and emphasizing the need for segregation analyses and functional studies to clarify their potential clinical relevance.

From a clinical perspective, the presence of high-risk features in two probands, namely implantable cardioverter-defibrillator implantation and a strong family history of sudden cardiac death, highlights the importance of vigilant surveillance and individualized risk stratification in patients with *MYBPC3*-related HCM. These findings emphasize the value of integrating genomic information with established clinical risk markers to inform preventive strategies against malignant arrhythmias and SCD.

Finally, the admixed Native American, European, and African ancestry observed in this cohort underscores the importance of expanding genomic studies in underrepresented populations. A broader inclusion of diverse ancestries will be essential to improve variant interpretation, refine risk assessment, and advance understanding of the genetic architecture and clinical expression of hypertrophic cardiomyopathy across populations.

## Data Availability

The original datasets presented in this study are publicly available in a community-supported repository. The data can be accessed in the NCBI Sequence Read Archive (SRA) under accession number PRJNA1399500: https://www.ncbi.nlm.nih.gov/sra/PRJNA1399500.
